# ThermoSteg—Covert Channel for Microbolometer Thermographic Cameras

**DOI:** 10.3390/s21196395

**Published:** 2021-09-24

**Authors:** Krzysztof Sawicki, Grzegorz Bieszczad, Tomasz Sosnowski

**Affiliations:** Institute of Optoelectronics, Military University of Technology, 00-908 Warsaw, Poland; grzegorz.bieszczad@wat.edu.pl (G.B.); tomasz.sosnowski@wat.edu.pl (T.S.)

**Keywords:** steganography, hardware security, thermography, covert channel, thermal cameras

## Abstract

The article presents a new concept—steganography in thermography. Steganography is a technique of hiding information in a non-obvious way and belongs to sciences related to information security. The proposed method, called ThermoSteg, uses a modification of one of the parameters of the thermal imaging camera—integration time—to embed the signal containing hidden information. Integration time changing makes the microbolometer array heat up while reading the sensors. The covert information can be extracted from the stream of thermograms recorded by another thermal camera that observes the first one. The covert channel created with the ThermoSteg method allows the transmission of covert data using a thermal sensor as a wireless data transmitter. This article describes a physical phenomenon that is exploited by the ThermoSteg method and two proposed methods of covert data extraction, and presents the results of experiments.

## 1. Introduction

Information is now one of the essential goods, and information security mechanisms are developing very quickly. For most of the usage scenarios, the best way to secure data is to use cryptography. However, in some specific cases, another method can be used—steganography [1,2,3,4]. Steganography is a technique that enables data transmission in a hidden way. For steganography, the critical factor is to make the data ‘covert’, so that no one would be able to receive the information. This approach differs from cryptography’s approach, where the data can be received by virtually anyone, but cannot be decrypted by anyone except the authorised recipient.

**Figure 1:**
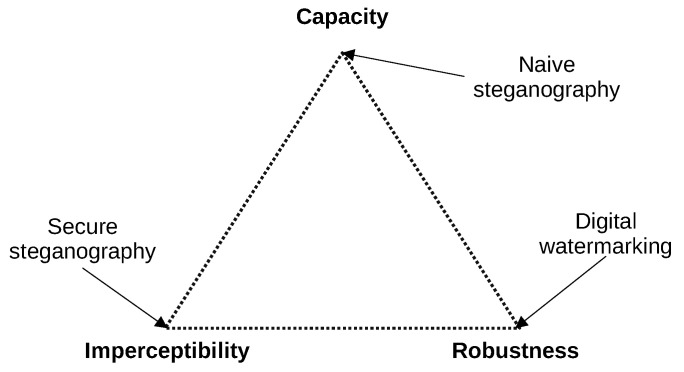
“Magic triangle of steganography.” [5,6].

Steganography creates so-called covert channels—communication channels where the data are transmitted in a hidden manner. The key element of these covert channels is the method of hiding data, which should be kept secret similar to the way in which the encryption key in cryptography is kept secret. Covert channels can be designed with different approaches: using unused bits in network protocol headers, modifying time-dependent parameters, or by using phenomena that are, in most cases, treated as unwanted or random. Many of the covert channels provide a bitrate smaller than tens of bits per second [4]. The very small bitrate makes the imperceptibility of the covert channel better according to the “magic triangle of steganography”. The covert channel should be imperceptible, robust and have a large capacity (bitrate). The rule of the “magic triangle” says that only two of these features can be achieved and the third feature will not be fully implemented (Figure 1) [5,6]. There are sophisticated covert channels that provide a bitrate on the order of tenths of a bit per second. The perfect example of such a covert channel is BitWhisper [7], where the authors achieved a bitrate of 8 bits per hour ($2.22\times 10^{-3}$ bps). Such low values make it impossible to use covert channels for audio or video transmission, but they are sufficient for signalling or the basic control of devices. Covert channels can be used when the fact of the presence of the communication should be kept secret, for example when the sensors that are placed in the enemy’s territory or on commonly used devices have a dual purpose, where secondary functionality is meant to be secret. Steganographic communication between sensors makes it harder to discover them. Steganographic communication might also be used as a part of so-called hardware trojans.

Covert channels and their usage are a popular research topic. The principles of covert channel creation were described in 1989 in Wolf [8]. Since then, many new and more sophisticated types of covert channels have been published. One of the most exploited fields is computer network steganography, where two types of covert channels can be distinguished—timing covert channels and storage covert channels. The first type uses the modulation of the interval between two network events, such as the beginning of packet transmissions in wireless channels [9] or adjusting silence periods in VoLTE transmission [10]. The other type, storage channels, exploits unused or partially used fields in network protocols, such as the Timestamp field in IEEE 802.11 Beacon frames [11]. Covert channels can also be embedded in other types of media, such as audio [12] and video [13] streams. It is also possible to create a storage covert channel in a phase drift of signals modulated with QAM modulations [14,15].

Such a broad selection of different covert channel types makes them an interesting subject for computer security teams. Covert channels can be a potential security breach and can be used for data leakage from network-isolated computer systems via ‘Air-Gap’. Many experiments of this type have been performed by Mordechai Guri [16,17,18] and other researchers [19,20,21]. For example, it is possible to use very low contrast or fast flickering images, which are invisible to human subjects, to transmit data using a computer display [22]. Another way is to use relatively cheap hardware to detect electromagnetic emissions from a USB [23]. Temperature can also be used as a medium for a covert channel. Two network-isolated computers can communicate via a covert thermal channel by stimulating CPU load on one computer as a transmitter and reading temperature sensors on the other computer as a receiver [7]. Covert channels created this way enable covert transmission with a bitrate of 8 bits per hour at a distance of up to 40 cm. Another example of hiding data in a non-obvious way with the use of thermal signals is presented in [24]. The method presented is based on the active heating of material by means of laser radiation. Unfortunately, the authors do not provide any bandwidth estimates of the proposed method.

This paper proposes a new type of covert channel that utilizes thermal cameras and their sensors to make steganographic communication possible. The covert channel is established between two thermal cameras, where one is acting as a transmitter, and the other can receive data. The steganographic transmission is possible thanks to the modification of some operational parameters of custom made microbolometric thermal cameras created for navigation systems [25]. The proposed covert channel can be classified as a timing channel because the data are hidden in the time characteristics of the thermal signal acquired from the part of the microbolometric sensors matrix that corresponds to the thermal image of the other’s camera sensor. The method uses a non-obvious way of modifying the camera’s parameters to enable transmission capabilities in microbolometric detectors.

## 2. Principles of the Method

**Figure 2:**
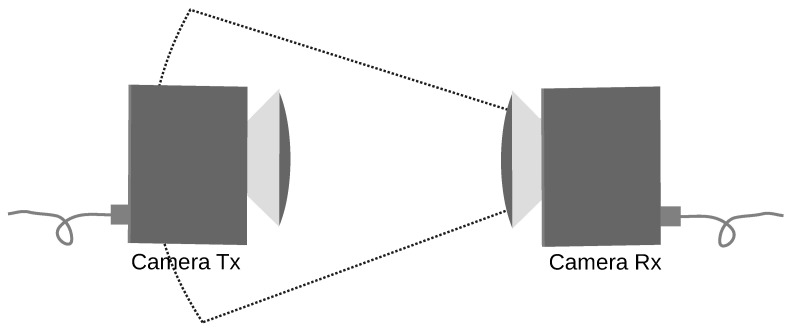
The concept of the covert channel using two thermal cameras, one for data transmission (Tx) and one for data reception (Rx).

The covert channel is a particular example of a telecommunication channel. In every communication channel, there must be a transmitter and a receiver. One camera acts as a hidden information transmitter (Tx) and the other as a receiver (Rx). Both cameras’ primary function is that of an ordinary thermal imaging camera. Such cameras could be an element of a security system or smart building infrastructure. In the proposed solution, two thermal imaging cameras are facing each other, as shown in Figure 2.

**Figure 3:**
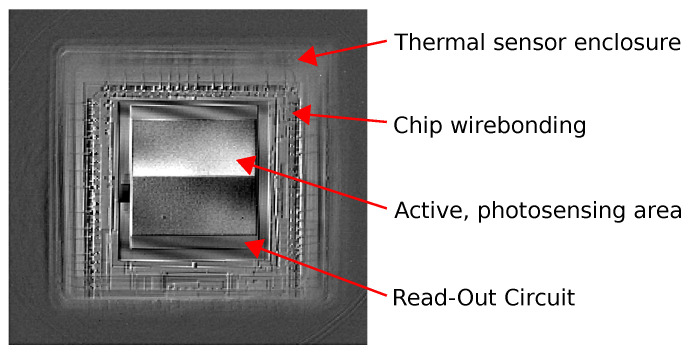
Thermal image of Micro80 detector array, made with a microscopic thermal camera.

The standard reading procedure needed to produce the thermal image is to retrieve data from the successive rows of thermal sensors. When the row of sensors in the matrix is read, an electric current flows through it. This current causes a temporary increase in the microbolometers’ temperature, according to Joule’s Law. This phenomenon in the microbolometer arrays is called self-heating [26]. Such a phenomenon is shown on a thermal image made with an FLIR SC7900VL and with a microscopic lens, presented in Figure 3.

The temperature change is significant enough to be sensed by a remote sensor, and for the exemplary case shown in the picture above, it reaches 2.2 °C in temperature amplitude. The secondary remote sensor, sensing the self-heating phenomenon, can be, for example, another thermal camera. This secondary thermal camera (Rx) facing the camera with a microbolometric sensor (Tx) can provide a thermal signal visible in the thermogram.

**Figure 4:**
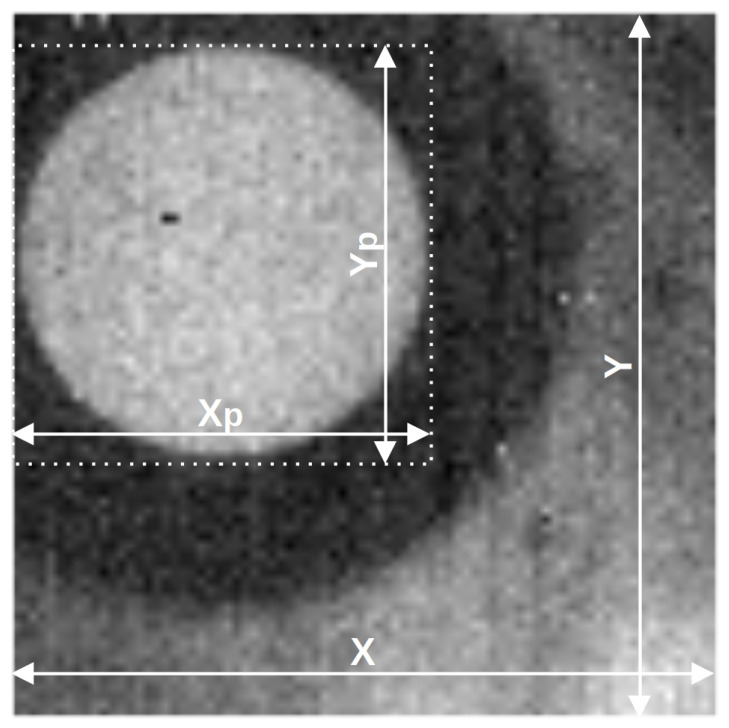
Image captured by Rx camera after nonuniformity correction. In the highlighted square, the Tx camera’s lens is visible.

In the area observed by the Rx camera, it is possible to distinguish the area (ROI—Region of Interest) in which the Tx camera lens is visible. Thanks to the fact that the thermal camera lens is transparent to the infrared spectrum, the thermal signal produced in the Tx camera by the self-heating phenomenon can be sensed remotely through the lens. This signal can be sensed by the Rx camera by observing the area that the Tx camera is occupying. The area is dependent on the Tx camera’s lens size, the Rx camera’s focal length and the distance between the cameras. An exemplary image of such an observed lens has a size of $X_{p}\cdot Y_{p}$ pixels and is marked in Figure 4, presenting the image obtained by the Rx Camera observing the Tx Camera. It should be noted that both cameras operate normally, which enables the registration of regular thermograms in both cameras.

### 2.1. Covert Data Coding and Embedding

**Figure 5:**
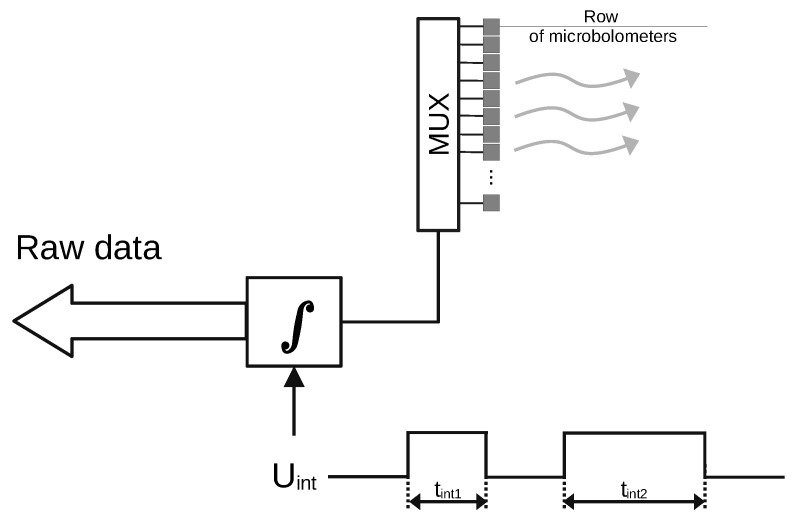
Data embedding scheme.

Coding of the covert data is performed by changing one of the main operational parameters of the microbolometer focal plane array—the integration time ($t_{i}$). Integration time regulates how long the measurement current from the Readout circuit flows through the row of bolometers in the array, which makes the self-heating phenomenon controllable. Reducing this time causes the current to flow shorter through the row in the bolometer, which results in less heating of the elements of this row; similarly, increasing $t_{i}$ will cause the elements of the currently read line to heat up more. These temperature differences can be detected with the use of the receiving camera. A detailed description of the operation of the readout circuit in the microbolometer array and its thermodynamic consideration are presented in [26]. The covert data are bivalently encoded using two different integration times $t_{i1}$ and $t_{i2}$. The hidden data embedding process is shown in Figure 5—the digital signal $U_{int}$, with the pulse width encoded by the covert data, controls the length of the integration process in the integration circuit. This paper considers the case when the covert data are binary. For this reason, the set of different integration times contains two values. It is possible to extend the method to use three or more different integration times to encode more values at a time.

### 2.2. Covert Data Reception—Amplitude Analysis

The ROI observation area contains $X_{p}\cdot Y_{p}$ pixels. Each observation is a subject of noise that is dependent on the NETD of the thermal camera and the optical path attenuation. The signal from the single detector can have an insufficient signal-to-noise ratio to extract subtle temperature changes in the observed microbolometric camera. To increase the signal-to-noise ratio, the spatial averaging is conducted in such a way that the average value of all $F_{n}$ pixels observed in the ROI is computed for each thermogram according to the formula:

(1) $$F\left(n\right)=\frac{\sum_{x=x_{1}}^{x_{2}}\sum_{y=y_{1}}^{y_{2}}p_{xy}}{\left(x_{2}-x_{1}\right)\left(y_{2}-y_{1}\right)},$$

where $p_{xy}$ is the value of the pixel with the coordinates (*x*,*y*) in the n-th thermogram, $x_{1}$ and $x_{2}$ are the numbers of the first and last columns of the analyzed area, while $y_{1}$ and $y_{2}$ are the numbers of the first and the last lines of the analyzed area.

**Figure 6:**
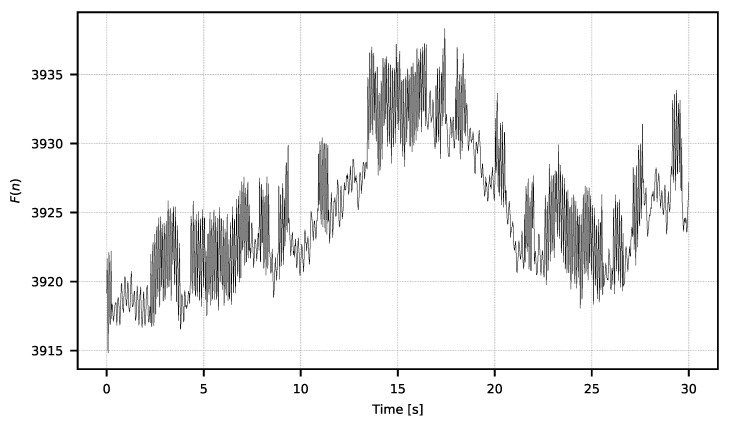
Exemplary *F* signal exhibiting strong 1/f noise.

This creates the signal $F=\{F_{0},F_{1},\ldots F_{N}\}$ of values from the averaged pictures, which constitutes the ROI’s temperature signal sampled with the camera operating frequency $f_{p}$. An example of the *F* signal is shown in Figure 6.

The *F* signal shows noticeable low-frequency noise resulting from the influence of external factors, that is, changes in the temperature of the camera’s surroundings. This noise has a typical 1/f spectral density and is very common in thermal sensors [27,28,29]. The spectral noise character and usable signal encoding scheme make it possible to separate one from another by means of a temporal filter. For further analysis, only the changes of higher frequency that interest us should be extracted; this is performed with the filter described by the equation:

(2) $$F^{\prime }\left(n\right)=F\left(n\right)-\frac{\sum_{i=n-w}^{n}F\left(i\right)}{w}\;for\;n=\left\{w,w+1,\ldots ,N\right\},$$

where F(n) is the n-th sample of the *F* signal, $F^{\prime }\left(n\right)$ is the n-th sample of the $F^{\prime }$ signal, *N* is the length of the *F* signal, *w* is the width of the applied window.

**Figure 7:**
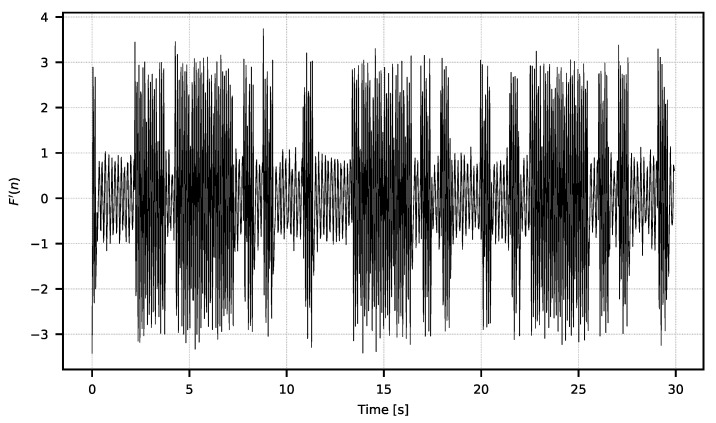
Signal $F^{\prime }$ after high-pass filtering.

The width of the window *w* should be selected experimentally according to the spectral characteristic of the low frequency noise. The exemplary resulting $F^{\prime }$ signal is shown in Figure 7.

**Figure 8:**
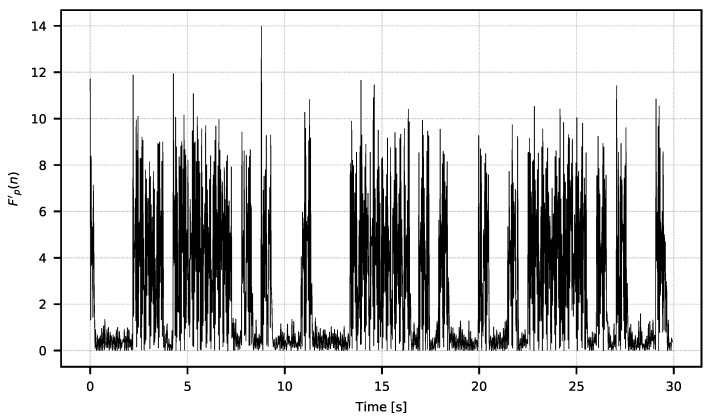
Signal $F_{p}^{\prime }$, which is the power of the filtered signal $F^{\prime }$.

In Figure 7, one can see the moments of the higher and lower amplitudes of the signal. The amplitude changes are the direct consequences of the integration time manipulation in the observed microbolometric array. Changes in signal amplitude can be easily estimated with power metrics according to the Formula (3). The resulting signal used for the analysis is shown in Figure 8.

(3) $$F_{p}^{\prime }\left(n\right)={\left[F^{\prime }\left(n\right)\right]}^{2}.$$

**Figure 9:**
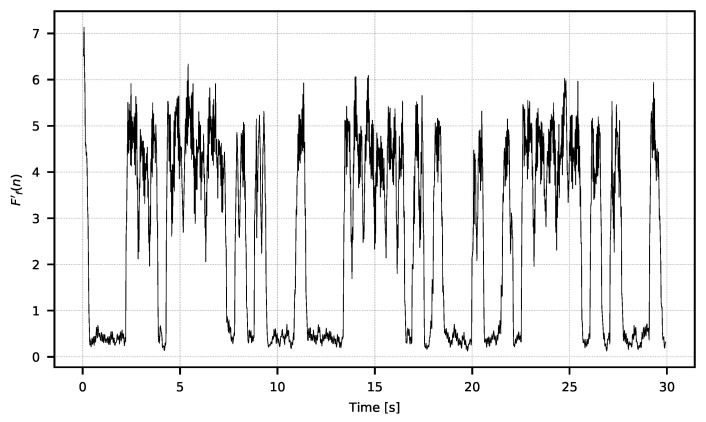
Signal $F_{f}^{\prime }$ averaged with window size $w^{\prime }=5$.

The $F_{p}^{\prime }$ signal still exhibits some noise, which is why it is then averaged over a temporal window of length $w^{\prime }$. The result of this averaging is the $F_{f}^{\prime }$ signal calculated with (4) and presented in Figure 9:

(4) $$F_{f}^{\prime }\left(n\right)=\frac{\sum_{i=n-w^{\prime }}^{n}F_{p}^{\prime }\left(i\right)}{w^{\prime }}.$$

The value of $w^{\prime }$ should be selected experimentally according to the characteristics of the signal received.

**Figure 10:**
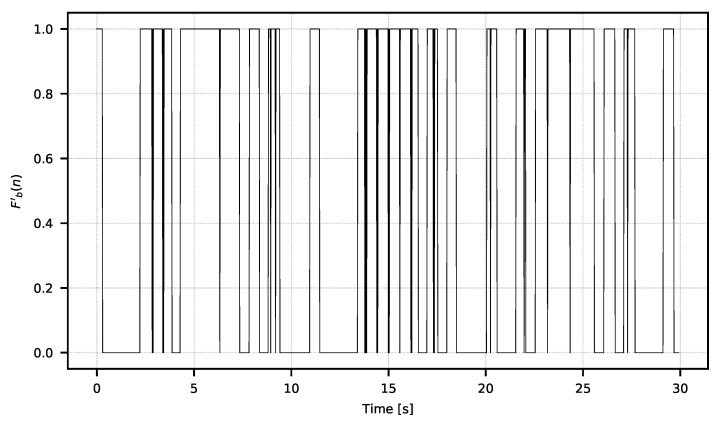
Received binary signal $F_{b}^{\prime }$.

In signal $F_{f}^{\prime }$, one can clearly see the characteristics of the binary waveform. To create a binary $F^{\prime }b$ signal, it is required to perform a threshold classification of the samples:

(5) $$F_{b}^{\prime }\left(n\right)=\left\{\begin{array}{c} 1\;when\;F_{f}^{\prime }\left(n\right)\geq \bar{F_{f}^{\prime }}\\ 0\;when\;F_{f}^{\prime }\left(n\right)<\bar{F_{f}^{\prime }}, \end{array}\right.$$

where $\bar{F_{f}^{\prime }}$ is the average value of all samples of the $F_{f}^{\prime }$ signal. An example of the $F_{b}^{\prime }$ signal is shown in Figure 10. The signal character corresponds to the signal used to supply the integration time changes in the Tx camera. The $F_{b}^{\prime }$ binary signal proves that hidden information is embedded in the *F* received signal.

### 2.3. Covert Data Reception—Variance Analysis

The algorithm’s effectiveness depends on the thermal amplitude of the received signal that is the consequence of the integration time values $t_{i}$, chosen to encode the thermal signal in the Tx camera. For a low signal amplitude caused, for example, by a large distance between the receiving and transmitting agents or lower lens transmission, amplitude demodulation can cause a high error rate. For such a situation, detection based on the signal variance analysis has been developed.

**Figure 11:**
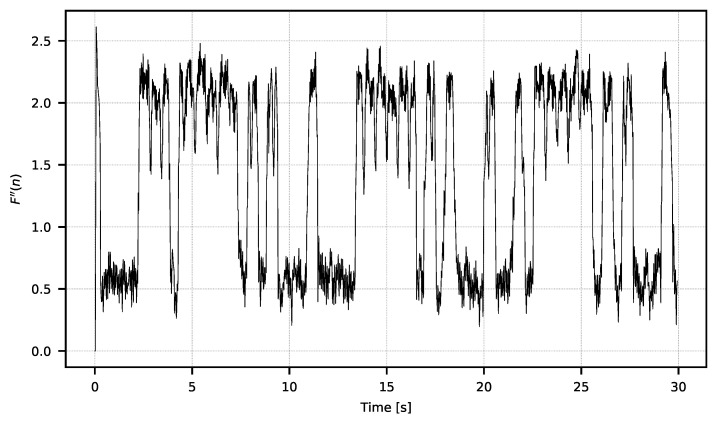
Signal $F^{\prime \prime }$ (6) calculated with $w_{s}=5$.

Having the $F=\{F_{0},F_{1},\;\ldots ,\;F_{n}\}$ signal calculated on the basis of (1), one can calculate the value of the $F^{\prime \prime }$ signal consisting of the value of the standard deviation of the *F* signal calculated in a temporal window with a width of $w_{s}$ according to Equation (6). Signal $F^{\prime \prime }\left(n\right)$ is also presented in Figure 11.

(6) $$F^{\prime \prime }\left(n\right)=\sqrt{\frac{1}{w_{s}}\sum_{i=n-w_{s}}^{n}{\left[F\left(i\right)-\left(\frac{1}{w_{s}}\sum_{j=n-w_{s}}^{n}F\left(j\right)\right)\right]}^{2}}.$$

**Figure 12:**
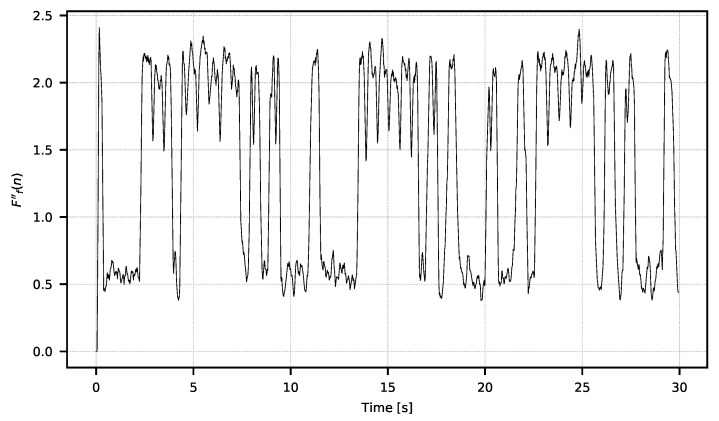
Signal $F_{f}^{\prime \prime }$ calculated with $w^{\prime \prime }=4$.

In Equation (6), $w_{s}$ denotes the width of the window in which the standard deviation is calculated and $F^{\prime \prime }\left(n\right)$ denotes the n-th sample of the signal $F^{\prime \prime }$. The $F^{\prime \prime }$ signal should be filtered (7) to obtain the $F_{f}^{\prime \prime }$ signal (Figure 12). Then, (8) should be classified, as a result of which the signal $F_{b}^{\prime \prime }$ will be obtained.

(7) $$F_{f}^{\prime \prime }\left(n\right)=\frac{\sum_{i=n-w^{\prime \prime }}^{n}F^{\prime \prime }\left(i\right)}{w^{\prime \prime }}$$

(8) $$F_{b}^{\prime \prime }\left(n\right)=\left\{\begin{array}{c} 1\;when\;F_{f}^{\prime \prime }\left(n\right)\geq \bar{F_{f}^{\prime \prime }}\\ 0\;when\;F_{f}^{\prime \prime }\left(n\right)<\bar{F_{f}^{\prime \prime }}. \end{array}\right.$$

### 2.4. Covert Data Extraction

The sampling frequency of $F_{b}^{\prime }$ and $F_{b}^{\prime \prime }$ binary signals is equal to the frequency of camera operation $f_{p}$. The covert data stream is sampled with a lower frequency, and every covert bit is conveyed by the specific number of equal value samples of $F_{b}^{\prime }$ and $F_{b}^{\prime \prime }$ signals. This number is defined as *B*:

(9) $$B=\frac{f_{p}}{W},$$

where *W* is the assumed hidden binary bit rate (e.g., 2 bps).

Based on this information, the binary sequence decoding process is performed. The algorithm written in Python finds the first change in the value of the sample input signal $F^{\prime }$ or $F^{\prime \prime }$ considering it the beginning of the covert bit. Then the algorithm averages the value of *B* samples counted from the beginning of the covert bit. If the mean value of these samples is greater than 0.5 then this is classified as the 1 covert bit. Otherwise, it is the 0 covert bit. Because some samples in the signals $F^{\prime }$ and $F^{\prime \prime }$ may be lost, the algorithm is able to synchronize to the binary string. The decoding algorithm is presented in Algorithm 1.
**Algorithm 1:** Decoding algorithm.  1:**procedure**decodeSignal(data, B, correction)  2:  decodedData←[]                ▹ Initialize an empty vector  3:  i←1  4:  bl←0  5:  recvBit←0  6:  **while** data[i] = data[0] **do**  7:    i←i+1  8:  **end while**  9:  **repeat**10:    **if** i+b>length(data) **then**11:      break12:    **end if**13:    **if** average(data[i:i+B])>0.5 **then**14:      recvBit←115:    **else**16:      recvBit←017:    **end if**18:    append(recvBit, decodedData)    ▹ Append recvBit to the end of the vector19:    i←i+B20:    **if** correction=1andi<length(data)anddata[i]≠recvBit **then**21:      offset←correction22:      **while** offset>0 **do**23:        **if** data[i]=data[i−offset] **then**24:          i←i−offset25:          break26:        **end if**27:        offset←offset−128:      **end while**29:    **end if**30:  **until** break31:  **return** decodedData32:**end procedure**

## 3. Experiments

### 3.1. Equipment

**Figure 13:**
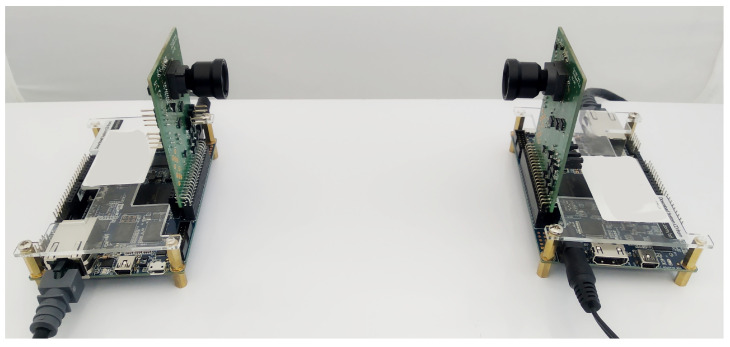
Camera setup for the method evaluation.

Two thermal imaging cameras based on FPGA Cyclone V and Lynred Micro80 matrices (80 × 80 pixels) [25] were used to perform the experiments. The camera matrices were positioned opposite each other at a distance of 14 cm, as shown in Figure 13. The cameras worked under the control of the GNU/Linux system and transmitted data using a dedicated protocol based on UDP over a network operating in the Gigabit Ethernet standard. The cameras generated thermograms with the frequency $f_{p}=44$ Hz, as it can be easily divided by two and by four. Further analysis was carried out using software written in Python on a PC.

### 3.2. Results and Discussion

**Figure 14:**
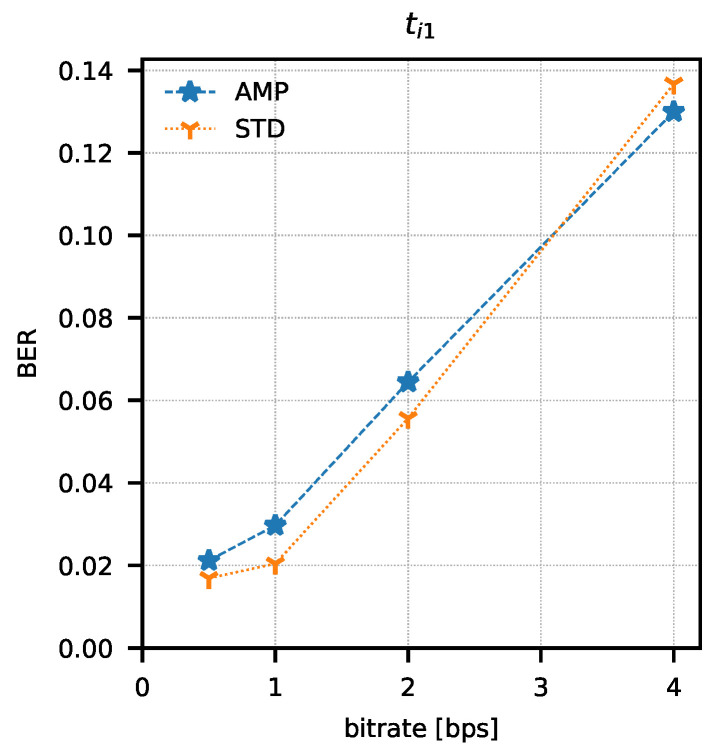
Bit error rate values as a function of bit rate and analysis method (AMP—amplitude analysis, STD—variance analysis) for $t_{i1}$ integration time set.

**Figure 15:**
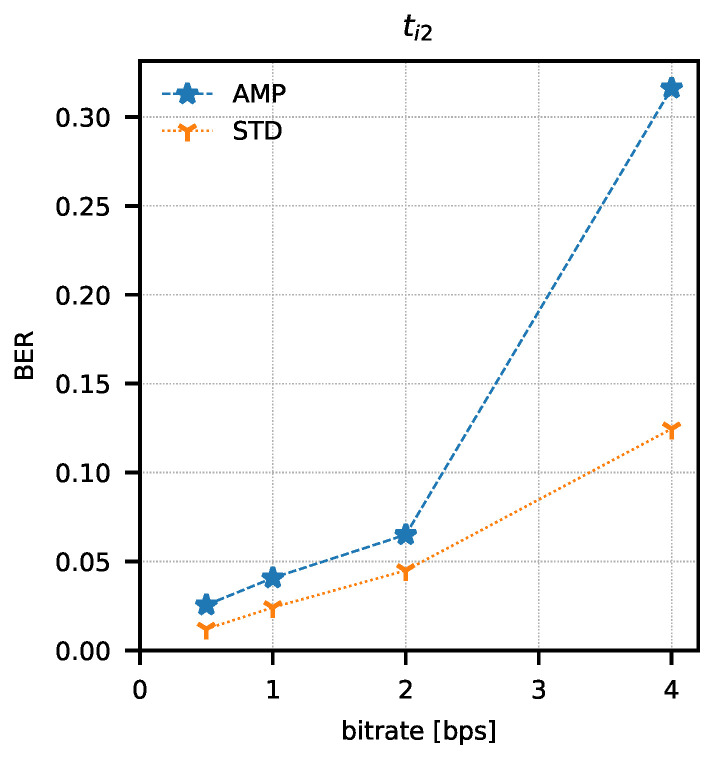
Bit error rate values as a function of bit rate and analysis method (AMP—amplitude analysis, STD—variance analysis) for $t_{i2}$ integration time set.

**Figure 16:**
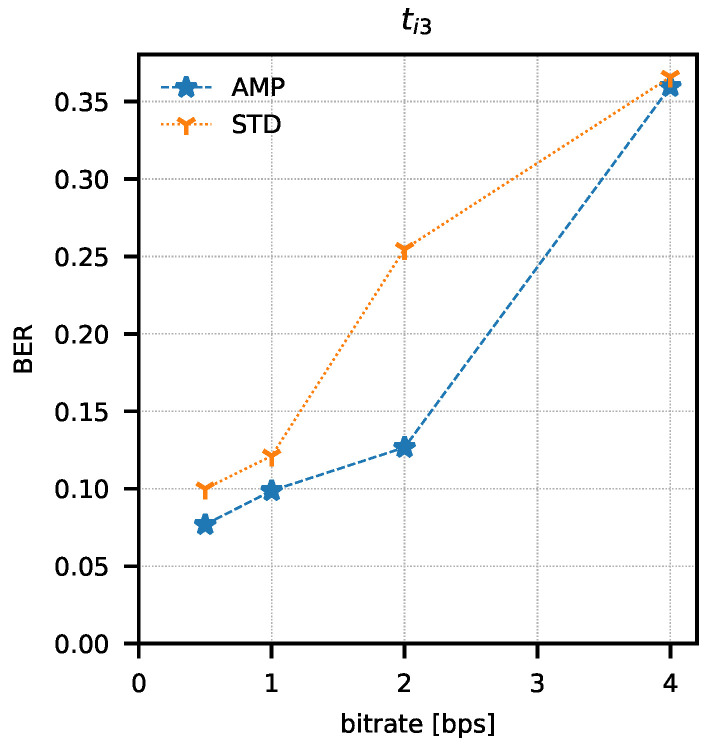
Bit error rate values as a function of bit rate and analysis method (AMP—amplitude analysis, STD—variance analysis) for $t_{i3}$ integration time set.

In order to determine the parameters of the method, attempts were made to transmit 1024 data packets of 10 bits each (every possible combination). Data packets were preceded by a one-bit preamble, which served as the start of the packet mark. The end of the data packet was also signalled with one stop-bit. The experiment was repeated for four different assumed binary bit rates W={0.5,1,2,4} b/s and three sets of integration times $t_{i1}=(82.05\;\mu$s; 328.21 μs), $t_{i2}=(123.08\;\mu$s; 287.18 μs), $t_{i3}=(164.10\;\mu$s; 246.15 μs). The bit error rate was determined using the amplitude analysis method and the amplitude variability analysis method for each set of integration times and binary bit rates. Any errors in the preambles were not taken into account when determining the bit error rate. Computed BER values are presented in Table 1 and Table 2. Graphical comparisons of the results are shown in Figure 14, Figure 15 and Figure 16.

Both methods of analysis produced similar results for the recorded transmissions. For $t_{i1}$ and $t_{i2}$, the variable analysis looks to be more promising as the BER values are almost two times better. The results create a chance for error-free transmission with the use of correction and detection codes in the information layer.

**Figure 17:**
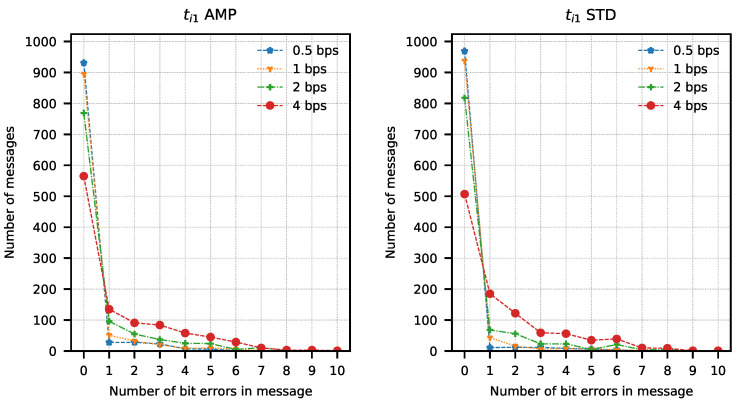
Number of bit errors in messages transmitted with $t_{i1}$ set calculated with amplitude analysis method (AMP) and amplitude variance analysis method (STD).

**Figure 18:**
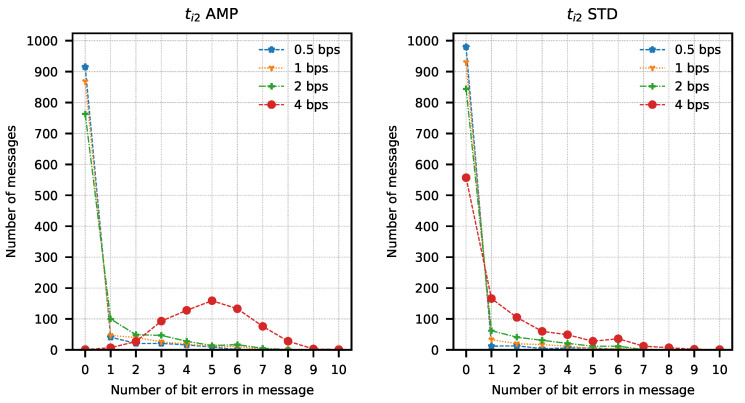
Number of bit errors in messages transmitted with $t_{i2}$ set calculated with amplitude analysis method (AMP) and amplitude variance analysis method (STD).

**Figure 19:**
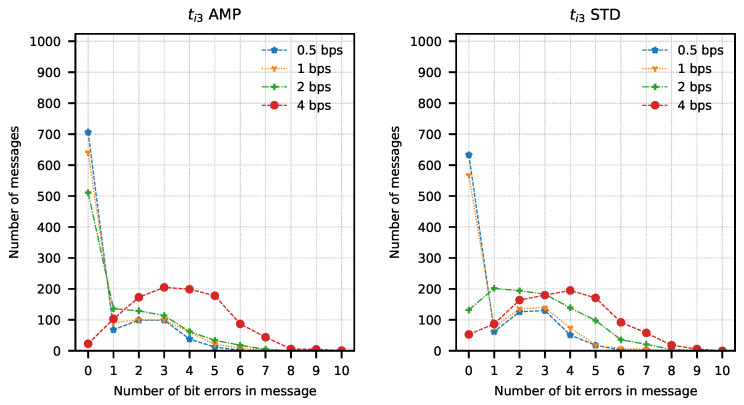
Number of bit errors in messages transmitted with $t_{i3}$ set calculated with amplitude analysis method (AMP) and amplitude variance analysis method (STD).

The analysis of error distribution in received messages was also performed. This analysis also shows how many messages have been received without errors. It is easy to observe that this distribution has the character of a Poisson distribution with an expected value equal to 0 in most cases. A different expected value was observed only with the highest bit rates, mostly with $t_{i3}$ integration times set. Results of the analysis are presented in Figure 17, Figure 18 and Figure 19. These figures show that most of the received messages were decoded with at most one error bit, which can be easily corrected with simple correction methods. They also show a slightly better number of correct messages decoded with amplitude variance analysis for $t_{i1}$ and $t_{i2}$ integration time sets.

**Figure 20:**
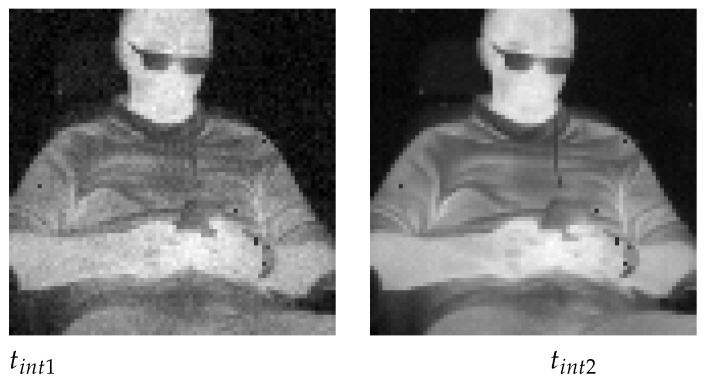
Two thermograms of the same scene acquired with different integration times ($t_{int1}=82.05\;\mu$s; $t_{int2}=328.21\;\mu$s).

Broadcasting information hidden by a thermal imaging camera does not disturb its primary task of producing a usable thermal image. In such a case, however, one should be aware of the need to introduce suitable correction of the thermograms, taking into account the changing values of the integration time. The difference between thermograms acquired with two different integration times is shown in Figure 20. With a shorter integration time, the dynamics of the output raw signal from the sensors are lower. Some degradation of thermal resolution (NETD) of the thermal camera will be present due to integration time manipulation. Because the change of the integration time still produces usable thermograms, there is a theoretical possibility of creating a full-duplex link with this method.

## 4. Conclusions

The hidden transmission methods do not ensure high bit rates. Hence, their scope of use is limited, for example, to the transmission of encryption keys or emergency device control commands. The proposed method can be used to create a communication channel between thermal imaging cameras transmitting, for example, encryption keys, performing camera authentication or the detection of unauthorized devices to prevent counterfeits or to eliminate rogue devices. This method can also be used to create a diagnostic interface with the thermal cameras that are mounted in inaccessible places such as a fire control system camera in a tank, or to transmit, for example, the coordinates of the transmitting camera, thanks to which the recorded thermograms can be supplemented with the information about the parameters of the monitored area. For such applications, bit rates of the order of single bits per second are sufficient. The undoubted advantage of the proposed method is its undetectability with the use of radio communication analysis equipment.

The proposed method has a limited operating range but can be used in dense networks of thermal imaging sensors, where the distances between the cameras are relatively small. Combined with the methods of infrared camera detection, this can increase the security of such networks.

## 5. Patents

The ThermoSteg method is a patent pending with application No. WIPO ST 10/C PL437673.

## Figures and Tables

**Table 1:** Bit error rate determined for the amplitude analysis method.

	$t_{\mathrm{int}}$	$t_{i1}$	$t_{i2}$	$t_{i3}$
*W*				
0.5 b/s		2.12%	2.55%	7.69%
1 b/s		2.97%	4.07%	9.87%
2 b/s		6.45%	6.50%	12.67%
4 b/s		13.00%	31.62%	35.97%

**Table 2:** Bit error rate determined for the amplitude variance analysis method.

	$t_{\mathrm{int}}$	$t_{i1}$	$t_{i2}$	$t_{i3}$
*W*				
0.5 b/s		1.69%	1.20%	10.00%
1 b/s		2.04%	2.41%	12.12%
2 b/s		5.57%	4.49%	25.47%
4 b/s		13.67%	12.46%	36.58%

## Data Availability

No data available.
